# Stillbirth and neonatal mortality in Jordan: findings from Jordan stillbirths and neonatal deaths surveillance system

**DOI:** 10.1186/s12978-025-01993-4

**Published:** 2025-05-31

**Authors:** Yousef Khader, Nihaya Al-Sheyab, Mohammad S. Alyahya, Ashley Azzam, Khulood K. Shattnawi, Ahmad Yosuf Abu Dalou, Ziad El-Khatib

**Affiliations:** 1 Department of Public Health, Faculty of Medicine, Jordan University of Science and Technology, P.O. Box 3030, Irbid, 22110 Jordan; 2 Department of Allied Medical Sciences, Faculty of Applied Medical Sciences, Jordan University of Science and Technology, P.O. Box 3030, Irbid, 22110 Jordan; 3 Department of Health Management and Policy, Faculty of Medicine, Jordan University of Science and Technology, P.O. Box 3030, Irbid, 22110 Jordan; 4 Faculty of Medicine, Yarmouk University, Irbid, Jordan; 5 Maternal & Child Health Nursing Department, Faculty of Nursing, Jordan University of Science and Technology, P.O. Box 3030, Irbid, 22110 Jordan; 6 Department of Anthropology, Faculty of Archaeology and Anthropology, Yarmouk University, Irbid, Jordan; 7 Medical University of Vienna, Vienna, Austria

**Keywords:** Neonatal mortality, Stillbirth, Risk factors, Causes, Surveillance

## Abstract

**Background:**

Stillbirth and neonatal mortality declined significantly in high- and some middle-income countries since 2000 because of the significant improvements in obstetric and neonatal care. Yet, stillbirth and neonatal mortality rates remain high in many low- and middle-income countries. The main reason for low progress in reducing such stillbirths and neonatal deaths in Jordan is the scarcity of data on causes and contributing factors leading to these deaths. This study aimed to determine the rates, causes, and risk factors of stillbirth and neonatal mortality in Jordan.

**Methods:**

An electronic stillbirth and neonatal deaths surveillance system was established in five large hospitals in Jordan. Anonymized data on all births, stillbirths, neonatal deaths, and their causes during the period May 2019–December 2020 were exported from the system and analyzed.

**Results:**

A total of 29,592 women gave birth to 31,106 babies during a period of 20 months in the selected hospitals. The stillbirth rate was 10.5 per 1000 total births, the neonatal death rate was 14.2 per 1000 live births, and the perinatal death rate was 21.4 per 1000 total births. Of all neonatal deaths, 29.4% died within the first day of life and 77.8% died during the first week of life. For neonatal deaths that occurred pre-discharge, the leading causes of death were respiratory and cardiovascular disorders (35.0%), low birth weight and prematurity (32.7%), and congenital malformations, deformations, and chromosomal abnormalities (19.5%). Almost one third of stillbirths had an unspecified cause of death (33.3% of antepartum stillbirths and 28.9% of intrapartum stillbirths). Intrauterine hypoxia was responsible for 27.4% of antepartum stillbirths and 13.2% of intrapartum stillbirths. Congenital malformations, deformations, and chromosomal abnormalities contributed to 18.1% of antepartum stillbirths and 34.2% of intrapartum stillbirths.

**Conclusions:**

Several identified maternal and/or fetal conditions that contributed to stillbirths and/or neonatal deaths in Jordan are preventable. Focused care needs to be directed to high-risk pregnant women and neonates with low birthweight and respiratory problems.

**Supplementary Information:**

The online version contains supplementary material available at 10.1186/s12978-025-01993-4.

## Plain language summary

While some countries have seen a drop in stillbirth and neonatal deaths due to better medical care, Jordan still struggles with high rates. The lack of detailed information about why these deaths happen has slowed progress there. The study aimed to figure out how often these deaths occur, why they happen, and what factors increase the risk in Jordan. To do this, we looked at data from five big hospitals in Jordan between May 2019 and December 2020. We found that out of 31,106 babies born to 29,592 women, there were 10.5 stillbirths per 1000 births and 14.2 neonatal deaths per 1000 births. Most neonatal deaths occurred in the first week, mainly due to issues like breathing and heart problems, low birth weight, and birth defects. About a third of stillbirths didn’t have a specific cause listed. The study showed that while the rates of these deaths have stayed about the same in Jordan, some reasons behind them can be prevented. We suggest focusing on providing specialized care for high-risk pregnant women and babies born with low birth weight or breathing issues to help lower these numbers.

**Supplementary Information:**

The online version contains supplementary material available at 10.1186/s12978-025-01993-4.

## Résumé

**Contexte:**

La mortinatalité et la mortalité néonatale ont considérablement diminué dans les pays à revenu élevé et dans certains pays à revenu intermédiaire depuis 2000 en raison des améliorations significatives apportées aux soins obstétricaux et néonatals. Pourtant, les taux de mortinatalité et de mortalité néonatale restent élevés dans de nombreux pays à revenu faible et intermédiaire. La principale raison des faibles progrès réalisés dans la réduction de ces mortinatalités et de ces décès néonataux en Jordanie est la rareté des données sur les causes et les facteurs contributifs conduisant à ces décès. Cette étude visait à déterminer les taux, les causes et les facteurs de risque de mortinatalité et de mortalité néonatale en Jordanie.

**Méthodes:**

Un système électronique de surveillance des mortinaissances et des décès néonatals a été établi dans cinq grands hôpitaux de Jordanie. Des données anonymisées sur toutes les naissances, mortinaissances, décès néonataux et leurs causes au cours de la période de mai 2019 à décembre 2020 ont été exportées du système et analysées.

**Résultats:**

Au total, 29 592 femmes ont donné naissance à 31 106 bébés sur une période de 20 mois dans les hôpitaux sélectionnés. Le taux de mortinatalité était de 10,5 pour 1 000 naissances totales, le taux de mortalité néonatale était de 14,2 pour 1 000 naissances vivantes et le taux de mortalité périnatale était de 21,4 pour un total de 1 000 naissances. Sur l’ensemble des décès néonatals, 29,4 % sont décédés au cours du premier jour de vie et 77,8 % au cours de la première semaine de vie. Pour les décès néonataux survenus avant la sortie de l’hôpital, les principales causes de décès étaient les troubles respiratoires et cardiovasculaires (35,0 %), le faible poids à la naissance et la prématurité (32,7 %), ainsi que les malformations congénitales, les déformations et les anomalies chromosomiques (19,5 %). La cause de décès n’était pas précisée pour près d’un tiers des mortinaissances (33,3 % des mortinaissances antepartum et 28,9 % des mortinaissances intrapartum). L’hypoxie intra-utérine était responsable de 27,4 % des mortinaissances antepartum et de 13,2 % des mortinaissances intrapartum. Les malformations congénitales, les déformations et les anomalies chromosomiques ont contribué à 18,1 % des mortinaissances antepartum et à 34,2 % des mortinaissances intrapartum.

**Conclusions:**

Plusieurs pathologies maternelles et/ou fœtales identifiées qui ont contribué aux mortinaissances et/ou aux décès néonataux en Jordanie sont évitables. Des soins ciblés doivent être dispensés aux femmes enceintes et aux nouveau-nés à haut risque présentant un faible poids à la naissance et des problèmes respiratoires.

## Background

Stillbirth and neonatal mortality declined significantly in high- and some middle-income countries since 2000 because of the significant improvements in obstetric and neonatal care. [1] Yet, stillbirth and neonatal mortality rates remain high in many low- and middle-income countries (LMICs) [2, 3] and almost 98% of stillbirths currently occur in these countries [4, 5].

In Jordan, neonatal mortality rates declined slowly in the last five decades, from 19 deaths per 1000 live births in the late 1980s to 14.9 deaths per 1000 live births in 2019 [6]. The trend in stillbirths, on the other hand, is hard to assess because stillbirths are not registered or reported in Jordan. Nevertheless, the Jordan Perinatal and Neonatal Mortality study reported that 74% of neonatal deaths and 64.8% of stillbirths are preventable or possibly preventable if high quality care is available [7].

The main reason for low progress in reducing such deaths is the scarcity of data on the causes and contributing factors leading to these deaths. In an attempt to overcome this problem, a secure electronic Jordan stillbirths and neonatal deaths surveillance system (JSANDS) was developed, established, and pilot-tested in five large hospitals in Jordan in May 2019 to collect, organize, analyze, and disseminate reliable data on births, stillbirths, and neonatal deaths and their causes [8–11]. One qualitative study using focus group discussions explored healthcare professionals’ and users’ perceptions of JSANDS usability in the five participating hospitals. Users found the system’s data useful, simple, and accurate, but identified areas for technical enhancement [8]. Moreover, an assessment conducted 18 months post-implementation of JSANDS revealed its commendable performance in registering stillbirths and neonatal deaths along with their causes [9]. A total of 270 healthcare professionals using JSANDS participated in this study and evaluated the system’s performance. The system users rated the usefulness of JSANDS as excellent (percentage score = 85.6%). The overall acceptability, flexibility, stability, and representativeness were also rated excellent (percentage score > 80%). The overall simplicity was scored good (percentage score = 75.4%). All participants were trained on JSANDS and used it in the past 12 months. Of the 270 respondents, 219 (86.2%) reported that they intend to continue using the JSANDS system to register neonatal deaths and stillbirths in the future. All variables in JSANDS had complete data with no missing values.

Two previous studies utilized data from JSANDS and determined the rate, risk factors, and causes of stillbirths and neonatal mortality in Jordan [10, 11]. The data used by these two studies were limited to the period of August 2019–January 2020, in which 10,328 registered births, 102 stillbirths, and 144 neonatal deaths were analyzed.

The current study used JSANDS data registered from May 2019 to December 2020 to provide accurate and more representative estimates of stillbirth and neonatal death rates as well as associated risk factors and causes. Better understanding the risk factors and causes of stillbirths and neonatal deaths using a large number of deaths over a longer period can contribute to improving the health care system and quality of care and ultimately preventing future deaths.

## Methods

### Study design and population

A retrolective cohort study using administrative data was conducted. All births, stillbirths, and neonatal deaths that occurred in five selected hospitals from May 2019 to December 2020 were registered in JSANDS. The five hospitals including one university teaching hospital, three Ministry of Health hospitals, and one private hospital were selected for pilot testing of the JSANDS. The criteria for selection of hospitals and JSANDS usability and functionality were described in previous studies [8–12]. All data on births, stillbirths, and neonatal deaths registered in JSANDS during the study period were retrieved and analyzed.

### Variables

The data entry sheet encompassed various variables including sociodemographic and obstetric characteristics of the mothers including age, education, employment status, income, gestational age, mode of delivery, and multiplicity. Mother’s age values were initially inputted as numbers and subsequently categorized during analysis into < 20 or 20–35 or > 35 year. Similarly, gestational age values were initially inputted as numbers and subsequently categorized during analysis into preterm or full-term births. Education levels were recorded as either “Diploma or less” or “Bachelor or higher”. Employment status data were captured as either “Housewife” or “Employed”, while income data were captured as either “≤ 500 JD” or “> 500 JD”. Mode of delivery information was entered as either “Normal” or “Cesarean section”, and multiplicity data were entered as “Singleton”, “Twin”, or “Triplet or more”. Other data included information on births’ characteristics including birth weight and status (born alive, stillbirth, and neonatal death). Causes of antepartum and intrapartum stillbirths and causes of neonatal deaths that occurred pre-discharge and post-discharge were also retrieved from JSANDS.

### Definitions

The definitions of stillbirths and neonatal deaths used in this study were based on the international standards set by the World Health Organization [13]. Neonatal mortality was defined as any death that happened at or after 24 gestational weeks within the first 28 days of life. The neonatal mortality rate was calculated as the number of neonatal deaths per 1000 live births (LB). Stillbirth was defined as any fetal death that occurred at or after 24 gestational weeks. Stillbirths were categorized as antepartum (deaths occurring before labor) and intrapartum stillbirths (deaths that occur after the onset of labor but before birth). The stillbirth rate was calculated as the number of stillbirths per 1,000 total births. Fetal weight percentile was determined based on birth weight and fetal age. Fetal growth was categorized as small for gestational age if < 10th percentile, appropriate for gestation if between 10th and 90th percentile, or large for gestation if > 90th percentile. Finally, to ensure comparability of stillbirth and neonatal mortality rates between different providers and to allow for international comparisons, births less than 24^+0^ weeks of gestation and terminations of pregnancy were not registered at the JSANDS.

### Identification of causes of death

Causes of neonatal deaths were identified according to the International Classification of Diseases-Perinatal Mortality (ICD-PM), which is part of the 10th version of the International Classification of Diseases (ICD-10) [13]. Training was held in the five hospitals for all healthcare providers on how to assign the cause of death. The attending physician has the primary responsibility to assign the cause of death and write the ICD-10 code accordingly. The ICD-10 codes were used to provide a unified language for reporting and monitoring diseases allowing a standardized comparison and sharing of data amongst the five hospitals. To assign the cause of death, the main disease or condition in the fetus or infant was identified and registered first and then any other diseases or conditions in the fetus or infant were identified, if any. Second, the main maternal disease or condition affecting the fetus was reported. The “most important” maternal disease or condition affecting the fetus that made the greatest contribution to fetal or neonatal death was reported. Other maternal diseases or conditions affecting the fetus, if any, were also reported. As there is no uniform protocol to evaluate and classify stillbirths in Jordan, malformations and chromosomal abnormalities were determined based on the attending obstetrician and neonatologist’s judgments of the obvious stillbirth malformations based on physical examination. According to the hospital’s protocol for placental examination, visual examinations of the placentas and umbilical cords were performed. In addition, a detailed summary of every stillbirth and neonatal death was discussed in monthly death review committee meetings attended by obstetricians, neonatologists, and senior midwives and nurses.

### Ethical approvals

The study was approved by the Institutional Review Board at Jordan University of Science and Technology (Ethical approval number 20170033). Permission from the Ministry of Health was granted to access the data and use it for research purposes. Informed consent was obtained from each participant before registration in JSANDS. To ensure data confidentiality, the data were exported without identifying information, such as the name or phone number.

### Data analysis

Data were described using means and standard deviations for continuous variables and rates and percentages for categorical data. The stillbirth and neonatal death rates were calculated and reported according to socio-demographic and clinical characteristics and compared using the Chi-square test. Multivariate analysis using binary logistic regression was used to determine factors associated with stillbirth and neonatal and perinatal death rates. A backward stepwise selection method was used to select the variables to be included in the regression model. A p-value of less than 0.05 was considered statistically significant. Data were described and analyzed using SPSS version 24 (IBM Corp. Released 2016. IBM SPSS Statistics for Windows, Version 24.0. Armonk, NY: IBM Corp.).

## Results

### Women and birth characteristics

A total of 29,592 women gave birth to 31,106 babies during the period from May 2019 to December 2020. Table 1 shows the sociodemographic characteristics of women. More than three quarters of women (78.3%) were in the age category of 20–35 years, 4.7% and 16.9% were below 20 and above 35 years of age, respectively. Of those, 88.3% were Jordanians and 11.7% were non-Jordanians. Only 9.9% were employed and the rest were housewives. Only 26.3% of women had a university education and 16.6% had income > 500 JD (1 JD = 1.4 US $). About 9.2% of deliveries were preterm. Of all births, 9.8% were born with low birth weight, 1.2% with very low birth weight, and 1.0% with extremely low birth weight. About 10.2% were born small for gestational age and 27.4% were born large for gestational age.

**Table 1:** Sociodemographic characteristics of women who gave birth from May 2019 to December 2020

Variables	n	%
Mother’s age (year)		
< 20	1405	4.7
20–35	23,180	78.3
> 35	5007	16.9
Hospital at birth		
Public hospital	19,752	66.7
Private hospital	5908	20.0
Teaching hospital	3932	13.3
Nationality		
Jordanian	26,139	88.3
Syrian refugees	3453	11.7
Mother’s employment		
Housewife	26,649	90.1
Employed	2943	9.9
Mother’s education		
Diploma or less	19,003	70.9
Bachelor or higher	7789	29.1
Income (JD = 1.40 US$)		
≤ 500 JD	22,988	82.4
> 500 JD	4916	17.6
Multiplicity		
Singleton	28,215	95.3
Twin	1274	4.3
Triplet or more	103	0.3
Gestational age		
Full term	26,873	90.8
Preterm	2719	9.2
Mode of delivery		
Normal	14,989	50.7
Cesarean section	14,603	49.3

### Stillbirth and neonatal mortality rates

Of all births, 326 were stillbirths (288 antepartum stillbirths and 38 intrapartum stillbirths), 436 died during the neonatal period (399 died pre-discharge and 37 died post-discharge), and 30,344 babies stayed alive after 28 days of birth. Of all neonatal deaths, 29.4% died within the first day of life. In total, 77.8% died during the first week of life (before 7 days of life).

Overall, the stillbirth rate was 10.5 per 1000 total births, the neonatal death rate was 14.2 per 1000 live births, and the perinatal death rate was 21.4 per 1000 total births. The death rates according to socio-demographic, obstetric, and clinical characteristics are shown in Table 2 and Table 3. Birth weight proportionate and specific mortality rates are shown in Table 4.

**Table 2:** Stillbirth, neonatal death, and perinatal death rates according to women’s socio-demographic characteristics

Variables	Stillbirth			Neonatal death			Perinatal death		
	n	Rate per 1000 TB	p-value*	n	Rate per 1000 LB	p-value*	n	Rate per 1000 LB	p-value*
Mother’s age			0.003			0.109			0.001
< 20	24	16.0		28	19.0		46	30.7	
20–35	231	9.5		326	13.5		484	19.8	
> 35	71	13.6		82	15.9		135	25.9	
Health sector			0.117			< 0.001			< 0.001
Public hospital	219	10.6		378	18.5		516	25.0	
Private hospital	53	8.6		19	3.1		68	11.0	
Teaching hospital	54	12.7		39	9.3		81	19.1	
Nationality			0.003			< 0.001			< 0.001
Jordanian	271	9.9		360	13.2		549	20.0	
Refugees	55	15.1		76	21.2		116	31.9	
Mother’s occupation			0.814			0.005			0.037
Housewife	293	10.4		411	14.8		616	21.9	
Employed	33	10.9		25	8.3		49	16.2	
Mother’s education			0.688			< 0.001			< 0.001
Diploma or less	201	10.1		330	16.8		463	23.3	
Bachelor or higher	79	9.6		57	7.0		119	14.4	
Income (1 JD = 1.4 US $)			0.230			0.009			0.201
≤ 500 JD	251	10.4		354	14.8		532	22.1	
> 500 JD	64	12.3		52	10.1		100	19.2	

*P-values indicate the significance of differences in mortality rates based on women’s socio-demographic characteristics. TB: Total births, LB: Live births

**Table 3:** Stillbirth, neonatal death, and perinatal death rates according to obstetric and clinical characteristics

Variable	Stillbirth			Neonatal death			Perinatal death		
	n	Rate per 1000 TB	p-value*	n	Rate per 1000 LB	p-value*	n	Rate per 1000 LB	p-value*
Baby’s gender			0.096			0.042			0.963
Female	165	11.5		178	12.5		305	21.2	
Male	160	9.6		253	15.3		354	21.2	
Multiplicity			< 0.001			< 0.001			< 0.001
Singleton	257	9.1		323	11.6		507	18.0	
Twin	52	20.3		92	36.7		122	47.7	
Triplet or more	17	50.9		21	66.2		36	107.8	
Parity			0.024			0.006			0.001
Nulliparity	16	16.0		13	13.2		26	26.0	
Low multiparity (parity 1–3)	193	9.4		257	12.7		393	19.2	
Grand multipara parity (+ 4)	117	12.1		166	17.4		246	25.4	
History of neonatal death			< 0.001			< 0.001			< 0.001
No	284	9.3		404	13.4		597	19.6	
Yes	42	73.3		32	60.3		68	118.7	
History of stillbirth			< 0.001			0.364			< 0.001
No	208	8.2		347	13.9		479	19.0	
Yes	118	20.1		89	15.4		186	31.6	
Gestational age			< 0.001			< 0.001			< 0.001
Extremely preterm (less than 28 weeks)	45	238.1		96	666.7		122	645.5	
Very preterm (28 to 32 weeks)	52	131.0		107	310.1		139	350.1	
Moderate to late preterm (32 to 37 weeks)	108	40.0		117	45.1		197	72.9	
Full term	121	4.3		116	4.2		207	7.4	
Birth weight			< 0.001			< 0.001			< 0.001
Normal birth weight	110	4.0		108	4.0		192	7.0	
Low birth weight	115	37.6		130	44.2		218	71.3	
Very low birth weight	48	130.4		107	334.4		131	356.0	
Extremely low birth weight	53	164.6		91	338.3		124	385.1	
Weight for gestational age			< 0.001			< 0.001			< 0.001
Appropriate for gestational age	138	7.1		208	10.8		299	15.4	
Small for gestational age	129	40.6		155	50.8		245	77.1	
Large for gestational age	59	6.9		73	8.6		121	14.2	
Mode of delivery			0.785			< 0.001			< 0.001
Normal	160	10.3		141	9.2		264	17.0	
Cesarean section	166	10.6		295	19.1		401	25.7	

*P-values indicate the significance of differences in mortality rates based on women’s obstetric and clinical characteristics. TB: Total births, LB: Live births

**Table 4:** Birth weight proportionate and specific mortality rates in Jordan*

	Numerator	Denominator	Rate per 1000
Birth weight proportionate mortality rate			
Pre-pregnancy health fetal-infant mortality rate	144	31,106	4.6
Care during pregnancy fetal-neonatal mortality rate	243	31,106	7.8
Care during delivery fetal-neonatal mortality rate	30	31,106	1.0
Pre-discharge care fetal-neonatal mortality rate	310	31,106	10.0
Post-discharge care fetal-infant mortality rate	35	31,106	1.1
Birth weight specific mortality rate			
BWS feto-infant mortality rate for < 1500 g	144	3026	47.6
BWS feto-infant mortality rate for 1500–2499 g	400	3426	116.8
BWS feto-infant mortality rate for > 2500 g	218	27,358	8.0
BWS antepartum fetal mortality rate for > 2500 g	98	27,358	3.6
BWS inrapartum fetal mortality rate for > 2500 g	12	27,358	0.4
BWS pre-discharge mortality rate for > 2500 g	86	27,358	3.1
BWS post-discharge mortality rate for > 2500 g	22	27,358	0.8

*The birth weight specific mortality rate is a stratification of a newborn mortality rate by birth weight grouping. The rate is calculated by dividing the number of perinatal deaths among newborns of a predetermined birthweight group by the total number of births in that weight group. The birth weight proportionate mortality rate is calculated by dividing the number of perinatal deaths among newborns of a predetermined birth weight group by the total births

### Risk factors of stillbirths, neonatal deaths, and perinatal deaths

Table 5 shows the multivariate analysis of factors associated with stillbirths, neonatal deaths, and perinatal deaths. The odds of neonatal deaths were significantly higher among babies born in public hospitals (OR = 2.0, 95% CI: 1.3–3.0) and lower among babies born in private hospitals (OR = 0.3, 95% CI: 0.1–0.6) compared to those born in teaching hospitals. Neonatal deaths were significantly more common among males compared to females (OR = 1.3, 95% CI: 1.0–1.6). Twins and triplets or more were at higher risk of being born dead (OR = 2.1, 95% CI: 1.5–2.9 and OR = 4.4, 95% CI: 2.3–8.3, respectively) and dying during the neonatal period (OR = 3.4, 95% CI: 2.6–4.4 and OR = 6.5, 95% CI: 4.0–10.7, respectively) compared to singletons. The odds of stillbirths (OR = 2.7, 95% CI: 1.5–4.9) and perianal deaths (OR = 1.7, 95% CI: 1.1–2.7) were significantly higher among babies born to nulliparous women compared to those born to grand multipara women. History of stillbirth or neonatal death was associated with increased odds of stillbirth, neonatal and perinatal deaths. Being small for gestational age was associated with increased odds of stillbirth (OR = 3.7, 95% CI: 2.8–4.9), neonatal deaths (OR = 3.0, 95% CI: 2.3–3.8), and perinatal death (OR = 3.3, 95% CI: 2.7–4.0). The most important determinant of death was preterm delivery (OR = 24.2, 95% CI: 18.8–31.1 for neonatal death, OR = 11.1, 95% CI: 8.4–14.7 for stillbirth, and OR = 18.2, 95% CI: 14.9–22.2 for perinatal death).

**Table 5:** Multivariate analysis of factors associated with stillbirths, neonatal deaths, and perinatal deaths

Variables	Neonatal death				Stillbirth				Perinatal death			
	OR	95% CI		p-value	OR	95% CI		p-value	OR	95% CI		p-value
Mother’s age												
< 20	1.2	0.7	2.1	0.428	1.2	0.6	2.1	0.643	1.2	0.8	1.9	0.343
20–35	0.9	0.7	1.2	0.538	0.8	0.6	1.0	0.09	0.8	0.6	1.0	0.085
> 35	1				1				1			
Gender of newborn												
Male	1.3	1.0	1.6	0.019	0.9	0.7	1.1	0.284	1.1	0.9	1.3	0.374
Female												
Type of hospital												
Public hospital	2.0	1.3	3.0	0.001	1.1	0.7	1.6	0.726	1.5	1.1	2.0	0.012
Private hospital	0.3	0.1	0.6	< 0.001	0.7	0.4	1.1	0.112	0.6	0.4	0.8	0.002
Teaching hospital	1				1				1			
Nationality												
Syrian refugees	1.3	1.0	1.8	0.068	1.4	1.0	2.0	0.086	1.4	1.1	1.9	0.006
Jordanians	1				1				1			
Mother’s occupation												
Housewife	1.1	0.6	1.7	0.826	1.1	0.7	1.7	0.672	1.1	0.8	1.6	0.594
Employed												
Mother education												
Diploma or less	1.2	0.8	1.8	0.282	1.1	0.7	1.5	0.788	1.1	0.9	1.5	0.347
Bachelor or higher	1				1				1			
Income												
> 500 JD	1.1	0.7	1.6	0.697	1.4	0.9	2.0	0.099	1.2	0.9	1.6	0.235
≤ 500 JD	1				1				1			
Multiplicity												
Singleton	1				1				1			
Twin	3.4	2.6	4.4	< 0.001	2.1	1.5	2.9	< 0.001	2.7	2.2	3.4	< 0.001
Triplet or more	6.5	4.0	10.7	< 0.001	4.4	2.3	8.3	< 0.001	6.1	4.0	9.2	< 0.001
Parity												
Nulliparity	1.0	0.5	2.0	0.954	2.7	1.5	4.9	0.001	1.7	1.1	2.7	0.030
Low multiparity (parity 1–3)	0.8	0.6	1.0	0.036	1.1	0.8	1.4	0.603	0.9	0.7	1.1	0.322
Grand multipara (parity + 4)	1				1				1			
History of neonatal death	4.1	2.7	6.1	< 0.001	6.3	4.3	9.2	< 0.001	5.3	4.0	7.2	< 0.001
History of stillbirth	0.9	0.7	1.2	0.670	0.4	0.3	0.6	< 0.001	0.6	0.5	0.8	< 0.001
Small for gestational age	3.0	2.3	3.8	< 0.001	3.7	2.8	4.9	< 0.001	3.3	2.7	4.0	< 0.001
Preterm delivery	24.2	18.8	31.1	< 0.001	11.1	8.4	14.7	< 0.001	18.2	14.9	22.2	< 0.001

### Causes of neonatal deaths

Table 6 shows the causes of neonatal deaths that occurred at pre- and post-discharge from the hospitals. For neonatal deaths occurring pre-discharge, the leading causes of death were respiratory and cardiovascular disorders (35.0%), low birth weight and prematurity (32.7%), and congenital malformations, deformations, and chromosomal abnormalities (19.5%). Of the total deaths caused by respiratory and cardiovascular disorders, respiratory distress of newborns (78.3%) and pulmonary hemorrhage originating in the perinatal period (13.8%) were the main causes. Of the 77 neonatal deaths caused by congenital anomalies, congenital malformations of the heart contributed to 10.4%, congenital malformation syndromes affecting multiple systems contributed to 9.1%, congenital hydrocephalus contributed to 6.5%, encephalocele contributed to 6.5%, congenital malformations of lung contributed to 6.5%, and anencephaly and similar malformations contributed to 5.2%. For neonatal deaths occurring after discharge, congenital malformations, deformations, and chromosomal abnormalities (27.0%), respiratory and cardiovascular disorders (21.6%), and low birth weight and prematurity (18.9%) were the leading causes of death. Maternal conditions contributed to 24.3% of neonatal deaths. Of all neonatal deaths affected by maternal conditions, 56.3% of pre-discharge deaths and 66.7% of post-discharge deaths were affected by complications of the placenta cord and membranes.

**Table 6:** Cause of neonatal deaths occurred pre- and post-discharge

Causes of neonatal deaths	Neonatal death/ pre-discharge		Neonatal death/ post-discharge	
	n	%	n	%
Cause of death (Main disease or condition in fetus or infant) and ICD-PM code				
N1- Congenital malformations, deformations and chromosomal abnormalities	77	19.5	10	27.0
N2- Disorders related to fetal growth	4	1.0	2	5.4
N3- Birth trauma	2	0.5	0	0.0
N4- Complications of intrapartum events	25	6.3	0	0.0
N5- Convulsions and disorders of cerebral status	6	1.5	0	0.0
N6- Infection	8	2.0	5	13.5
N7- Respiratory and cardiovascular disorders	138	35.0	8	21.6
N8- Other neonatal conditions	5	1.3	3	8.1
N9- Low birth weight and prematurity (P07-Disorders related to short gestation and low birth weight not elsewhere classified)	129	32.7	7	18.9
N11- Neonatal death of unspecified cause	0	0.0	1	2.7
Main maternal disease or condition affecting fetus or infant				
M1- Complications of placenta cord and membranes (P02-Fetus and newborn affected by complications of placenta cord and membranes)	58	56.3	2	66.7
M2- Maternal complications of pregnancy (P01-Fetus and newborn affected by maternal complications of pregnancy)	33	32.0	0	0.0
M3- Other complications of labour and delivery (P03-Fetus and newborn affected by other complications of labour and delivery)	1	1.0	0	0.0
M4- Maternal medical and surgical conditions (P00-Fetus and newborn affected by maternal conditions that may be unrelated to present pregnancy)	11	10.7	1	33.3

* The causes of death was not established in 5 neonatal deaths

### Causes of stillbirths

Almost one third of stillbirths had unspecified causes of death (33.3% of antepartum stillbirths and 28.9% of intrapartum stillbirths) (Table 7). The acute antepartum event was responsible for 27.4% of antepartum stillbirths and the acute intrapartum event was responsible for 13.2% of intrapartum stillbirths. Congenital malformations, deformations, and chromosomal abnormalities contributed to 18.1% of antepartum stillbirths and 34.2% of intrapartum stillbirths. The most common congenital anomalies contributed to stillbirths included anencephaly and similar malformations (20.0%), congenital hydrocephalus (15.4%), and other congenital malformations of the brain (12.3%). Maternal conditions contributed to 33.7% of stillbirths. Of all stillbirths affected by maternal conditions, 46.5% of antepartum stillbirths and 72.7% of intrapartum stillbirths were affected by complications of the placenta cord and membranes.

**Table 7:** Causes of antepartum and intrapartum stillbirths

Stillbirths/antepartum			Stillbirth/intrapartum		
	n	%		n	%
Cause of death (Main disease/ condition in fetus) and ICD-PM code			Cause of death (Main disease/ condition in fetus) and ICD-PM code		
A1- Congenital malformations deformations and chromosomal abnormalities	52	18.1	I1- Congenital malformations deformations and chromosomal abnormalities	13	34.2
A2- Infection	3	1.0			
A3- Acute antepartum event	79	27.4	I3- Acute intrapartum event	5	13.2
A4- Other specified antepartum disorder	17	5.9	I5- Other specified intrapartum disorder	4	10.5
A5- Disorders related to length of gestation and fetal growth	24	8.3	I6- Disorders related to fetal growth	3	7.9
A6- Antepartum death of unspecified cause	96	33.3	I7- Intrapartum death of unspecified cause	11	28.9
Cause is not established	17	5.9	Cause is not established	2	5.3
Main maternal disease or condition affecting fetus			Main maternal disease or condition affecting fetus		
M1-Complications of placenta cord and membranes	46	46.5	M1-Complications of placenta cord and membranes	8	72.7
M2-Maternal complications of pregnancy	25	25.3	M2-Maternal complications of pregnancy	1	9.1
M3-Other complications of labour and delivery	6	6.1	M3-Other complications of labour and delivery	1	9.1
M4-Maternal medical and surgical conditions (P00-Fetus affected by maternal conditions that may be unrelated to present pregnancy)	22	22.2	M4-Maternal medical and surgical conditions P04-Fetus affected by noxious influences transmitted via placenta	1	9.1

## Discussion

This study analyzed JSANDS data on 326 stillbirths, 436 neonatal deaths, and 30,344 babies who stayed alive after 28 days of birth. The research team verified all deaths registered through the JSANDS with those documented on paper and electronic medical records in the five selected hospitals to avoid any missing deaths. We found only 1% inconsistency between deaths registered through the JSANDS and those registered on papers mainly due to some delays in entering the death case to the JSANDS. However, all deaths tend to be registered in the JSANDS within one day of the occurrence of death.

Our findings contribute to the current national literature about rates, causes, and risk factors of stillbirth and neonatal mortality in Jordan. Our findings are novel as they represent all births, stillbirths, and neonatal deaths that occurred from May 2019 until December 2020 across these five hospitals. Analyzing high quality and reliable data of this long period can provide precise estimates of the current situation in Jordan about rates, causes, and contributing factors of stillbirth and neonatal mortality.

The current study found that the stillbirth rate was 10.5 per 1000 total births, and this is higher than that in a recently published study that used the JSANDS data, but for a much shorter period (from August 2019 to January 2020) [11]. However, this rate was similar to that reported in a previous national study in 2012 [7] reflecting more improvements needed in maternal and child health care in the country to achieve even lower stillbirth rates that can be comparable to those in developed countries [14]. Nonetheless, our stillbirth rates are lower than those reported in 2019 in the Middle East and North Africa (12.6 per 1,000 total births) [15].

The neonatal death rate in the current study was 14.2 per 1,000 live births, and the perinatal death rate was 21.4 per 1000 total births. These rates are similar to those published in a recent study that also used the JSANDS data but for a much shorter period [1] and rates reported in an earlier national study but used a different cut-off point of gestational weeks (≥ 20 weeks) [7]. Between 1990 and 2017, the global neonatal mortality rate decreased by 51% [16] yet, the stability of the rate in countries with high neonatal mortality rates calls for intensified efforts in order to achieve the Sustainable Development Goal by 2030 [6].

In comparison with other Middle-Eastern countries, the UN Inter-Agency Group for Child Mortality Estimation estimated that the 2019 weighted average neonatal mortality rate was 12.3 in the Middle East, ranging from 3 in Bahrain and Qatar to 31 in Djibouti [17]. In addition, the current study reported that 29.4% of neonatal deaths occurred within the first day of life. This percentage is somehow lower than that reported by the WHO, in which around half of all neonatal deaths in LMIC occurred within 24 h of birth and one third in the first six hours after birth [18].

Our findings reveal that acute antepartum events (intrauterine hypoxia) were responsible for almost one-third of antepartum stillbirths whereas acute intrapartum events were responsible for 13.2% of intrapartum stillbirths. This is similar to the findings of a recently published study that used the data registered at JSANDS for six months [11]. Conversely, our findings showed that congenital malformations, deformations, and chromosomal abnormalities contributed to 18.1% of antepartum stillbirths which is slightly higher than the recently published local study [11]. This variation in rates between the two studies that used the JSANDS data is somehow expected as the current study used a large dataset for a longer period (19 months versus 6 months) that allowed more births and deaths to be registered in the JSANDS, hence more accurate findings are demonstrated.

Moreover, our findings showed that maternal conditions contributed to almost one-third of stillbirths, in which complications of the placenta, cord, and membranes were the most common causes of maternal conditions affecting the fetus. In an earlier national study that used the Neonatal and Intrauterine Death Classification according to Etiology classification, the main causes of stillbirths were also maternal diseases, followed by unexplained immaturity, congenital anomalies, unexplained antepartum stillbirths, obstetric complications, and placental abruption [19]. Importantly, our study distinguished between the cause of death and risk factors whereas previous national studies [7, 19] used different classifications of causes that did not separate causes from risk factors but rather were in the same category.

In LMICs, the majority of stillbirths occur unexpectedly, without a clear cause [20] because many factors may contribute to the cause of stillbirth. For example, maternal diabetes, hypertension, and other medical disorders comprised about 52.4% of total stillbirths in the Middle-Eastern population whereas intrauterine growth restriction and congenital anomalies were fetal factors contributing to stillbirths in the same population [21]. Regardless, late fetal deaths were more likely to be preventable or possibly preventable compared to early fetal deaths.

When it comes to the causes of neonatal deaths at pre-discharge reported in our study, the leading causes were respiratory and cardiovascular disorders, followed by low birth weight and prematurity, and congenital malformations. This study reported similar results to Al-Sheyab et al., congenital malformations, followed by respiratory and cardiovascular disorders, low birth weight, and prematurity as the leading causes of neonatal deaths at post-discharge [10]. Conversely, a previous national study reported that congenital anomalies were the main cause of neonatal death followed by unexplained immaturity [7]. It should be noted that in the current study, we segregated neonatal causes of death at pre-discharge from those at post-discharge but the previous national study [7] reported causes of neonatal death without separating those pre-discharge from those a post-discharge. Another possible reason for the variation in the main cause of neonatal death between the current study and the previous national study was the low percentage of neonatal deaths at post-discharge (8%) as the vast majority (92%) of neonatal deaths occurred at pre-discharge, which were mostly caused by cardiovascular and respiratory conditions. Taking this into consideration, it appears that the most legitimate cause of neonatal deaths in Jordan is cardiovascular and respiratory conditions.

Some of the causes and risk factors of perinatal mortality that were identified in the current study were also reported in the literature in developing countries. In LMICs, neonatal factors associated with increased mortality were congenital anomalies, resuscitation, male sex, hospitalization, lower birth weights, and gestational ages [22] whereas maternal factors were maternal death, previous stillbirth, nulliparity, abnormal fetal presentation, hypertensive disease, obstructed labor, and severe postpartum hemorrhage [22]. Similarly, a recent meta-analysis [22] found a significant decrease in perinatal mortality among women who gave birth to full-term and normal birth weight newborns and attended regular antenatal care but no impact by mode of delivery. Still, the relationship between perinatal mortality and mode of delivery, parity, and fetal gender requires additional research [23]. Another meta-analysis found a significant increase in perinatal mortality with young maternal age and short birth interval but was not influenced by women’s place of residence, low educational level, and household wealth index [24]. Alarmingly, about half of neonatal deaths in the Global Network sites occurred in infants born weighing ≥ 2500 g [22] with a mortality rate of 13.1 per 1000 live births, which is much higher than rates usually reported in high-income countries. Since many neonatal deaths should be preventable, attention to preventing mortality in these infants should have an important impact on the overall neonatal mortality rate [22].

When it comes to risk factors, our findings highlighted that the odds of neonatal and perinatal deaths were significantly higher among babies born in public hospitals and lower among babies born in private hospitals, when compared to teaching hospitals. One of the reasons that can explain higher odds of perinatal deaths in public hospitals is that women with complicated pregnancies and/or labor tend to seek public hospitals because of the relatively low cost and comprehensiveness of multidisciplinary medical care [25]. Another possible reason is the high demand for public maternity hospitals will ultimately lead to a higher number of births there and hence could increase the risk of medical errors due to work overload. More importantly, many Jordanian women choose to receive antenatal care at a private clinic but give birth in a public facility as they cannot afford to give birth in a private hospital [25]. This practice can lead to complicated labor and/or insufficient neonatal care due to a lack of maternal history during pregnancy at public maternity clinics. The WHO reported that poor quality of antenatal care can contribute to stillbirth [26]. Similarly, a wide coverage of antenatal care is promising in preventing or reducing stillbirths [27].

Similar to the findings of previous local studies [7, 10], the current study also found that twins or triplets were at much higher risk of being born dead or dying during the neonatal period compared to singletons. Also, the odds of stillbirths and perianal deaths were significantly higher among babies born to nulliparous compared to those born to grand multipara. Similarly, a history of stillbirth or neonatal death was associated with increased odds of stillbirth, neonatal, and perinatal deaths. Literature also showed that having stillbirth previously significantly increases the risk for recurrent stillbirth and other adverse outcomes in consecutive pregnancies [28].

In a qualitative study [29], healthcare providers identified key determinants of perinatal deaths in Jordan. Maternal, sociocultural, political, and health system-related factors were recognized as significant contributors. Factors such as ignorance, concealment of medical conditions, socioeconomic status, early marriage due to displacement, and health service-related issues were highlighted. Employing the “three-delay” model, a study investigated critical delays contributing to neonatal deaths and stillbirths in Jordan. Delays in recognizing the need for care, in seeking care, and in receiving care were identified, with specific modifiable factors like poor awareness, delayed antenatal care, and training inadequacies. Actions were initiated to address these delays and reduce preventable deaths [30].

The study findings have several implications for the prevention of neonatal deaths and stillbirths. The leading causes of neonatal deaths in Jordan encompass respiratory and cardiovascular disorders, low birth weight and prematurity, and congenital malformations. Foundational interventions crucial for managing conditions linked to prematurity include thermal care, breastfeeding support, infection prevention and management, and neonatal resuscitation. Of paramount importance is the provision of high-quality antenatal care. This entails identifying high-risk pregnancies and detecting complications related to childbirth. Effective management of these complications and ensuring appropriate referrals are pivotal aspects of antenatal care. In addition to antenatal care, other interventions play vital roles. Adequate quality of emergency obstetric care services is indispensable for addressing complications during childbirth promptly and effectively. Likewise, ensuring the availability of high-quality immediate newborn services is essential for providing timely interventions to newborns in need.

The current study has several strengths. First, all deaths occurring in any of the five hospitals were registered on the JSANDS within one day of the occurrence of death with less than 1% incongruence between the total number of deaths registered on papers and those registered through the JSANDS. This reflects the high accuracy and reliability of the JSANDS as well as the compliance and commitment of healthcare workers to register all births and deaths in JSANDS. Second, the JSANDS identified the exact cause of death and risk factors with a clear distinction between the two, whereas previous national studies reported causes and risk factors of death together. Third, to ensure comparability of stillbirth and neonatal mortality rates between different providers and to allow for international comparisons, births less than 24^+0^ weeks of gestation and terminations of pregnancy are not registered at the JSANDS. The current findings were based on the 18-month period of registered births, stillbirths, and neonatal mortality in the five hospitals; a longer period than the two recently published studies that analyzed only six months of the registered JSANDS data [10, 11].

Yet, the study has some limitations that need to be acknowledged. The JSANDS system did not include all hospitals in Jordan, and this could have limited the generalizability of the findings at a national level. However, the five selected hospitals were from three major Jordanian governorates, where the majority of births, stillbirths, and neonatal deaths occur. Two of these hospitals are only specialized in maternal and child health, and two of them are large university teaching, referral hospitals. We aim to utilize the data registered in the JSANDS to closely monitor the rate, causes, and risk factors of neonatal mortality and stillbirth in Jordan. Nevertheless, our findings are considered a reliable baseline for policymakers to develop and enforce policies and interventions to decrease preventable stillbirth and neonatal mortality in Jordan.

## Conclusions

Several identified maternal and/or fetal conditions that contributed to stillbirths and/or neonatal deaths in Jordan are preventable. Focused care needs to be directed to high-risk pregnant women and neonates with low birthweight and respiratory problems. Given the fact that the vast majority of stillbirths and early neonatal deaths- especially those occurring at pre-discharge- are under-reported in Jordan, capitalizing the health information systems to improve data registration can motivate healthcare providers and policymakers to quickly and specifically target interventions to reduce perinatal deaths.

## Supplementary Information

Additional file 1. The supplementary data file contains detailed data on sociodemographic, obstetric, and clinical characteristics of women who gave birth between May 2019 and December 2020 from Jordan stillbirths and neonatal deaths surveillance system, as well as the associated outcomes such as stillbirth, neonatal death, and perinatal death

## Data Availability

The datasets used and/or analyzed during the current study are available as a supplementary file.

## Abbreviations

BWS: Birth weight proportionate and specific
ICD-PM: International Classification of Diseases-Perinatal Mortality
JD: Jordan Dinar
JSANDS: Jordan stillbirths and neonatal deaths surveillance system
LMICs: Low-and- middle-income countries
OR: Odds ratio
SPSS: Statistical Package for Social Sciences
WHO: World Health Organization
